# Prevalence of chronic widespread pain in a population-based cohort of patients with spondyloarthritis – a cross-sectional study

**DOI:** 10.1186/s41927-018-0018-7

**Published:** 2018-04-05

**Authors:** Elisabeth Mogard, Ann Bremander, Elisabet Lindqvist, Stefan Bergman

**Affiliations:** 1 Department of Clinical Sciences Lund, Rheumatology, Lund University, Skane University Hospital, Lund, Sweden; 2 Department of Clinical Sciences Lund,Rheumatology, Lund University, Faculty of Medicine, Lund, Sweden; 3 School of Business, Engineering and Science, Rydberg Laboratory for Applied Science, Halmstad University, Halmstad, Sweden; 4 Spenshult Research and Development Center, Halmstad, Sweden; 5 Primary Health Care Unit, Department of Public Health and Community Medicine, Institute of Medicine, Sahlgrenska Academy, University of Gothenburg, Gothenburg, Sweden

**Keywords:** Spondyloarthritis, Ankylosing spondylitis, Undifferentiated spondyloarthritis, Chronic widespread pain, Sex

## Abstract

**Background:**

Chronic pain, regional or widespread, is a frequent and multidimensional symptom in arthritis. There is still limited information on chronic pain in spondyloarthritis, which is important to recognize for adequate diagnosis and treatment. Our objective was to study differences in prevalence of chronic widespread pain in two spondyloarthritis subgroups: ankylosing spondylitis (AS) and undifferentiated spondyloarthritis (USpA).

**Methods:**

A population-based postal survey involving questions on the duration, distribution, and intensity of pain was answered by 940 patients with AS (ICD-10 M45.9) or USpA (ICD-10 M46.1-0, M46.8-9). The patients were categorized as having chronic widespread pain, chronic regional pain, or no chronic pain, and prevalence estimates for the pain groups were calculated, including age- and sex-adjusted prevalence.

**Results:**

The prevalence of chronic widespread pain was 45.3% in AS vs. 49.3% in USpA, and that of chronic regional pain was 17.7% vs. 21.9% (*p* = 0.033). More women than men reported having chronic widespread pain (54.1% vs. 41.2%, *p* ≤ 0.001), while the sex distribution for chronic regional pain was equal. Reports of pain intensity were equal in AS and USpA, with no significant difference in pain intensity between women and men who had chronic regional pain or chronic widespread pain. In the multiple logistic regression analysis, chronic widespread pain was associated to female sex, being an ever-smoker, and having a higher body mass index, controlled for SpA subgroup and disease duration.

**Conclusions:**

The prevalence of chronic widespread pain in patients with AS and USpA is high, and with a female predominance, but with no difference in pain intensity between women and men. Chronic pain can complicate the clinical evaluation in patients with SpA, and highlights the need for a thorough clinical examination, including evaluation of inflammation and an accurate pain analysis, to individualize non-pharmacological and pharmacological treatment decisions.

## Background

Pain is a frequent and multidimensional symptom in patients with arthritis [1, 2]. Apart from the common inflammatory nociceptive pain, inflammation and nociceptive stimuli in peripheral and axial joints may also cause a heightened pain perception due to both peripheral sensitization, with nociceptors responding to light pressure and normal movement, and central sensitization with hyperexcitability of the neurons in the spinal cord [1, 3, 4]. This heightened pain perception could lead to a persistence of the pain and development of a chronic pain condition [5]. Chronic pain can be divided into chronic regional pain (CRP) and chronic widespread pain (CWP). CWP is usually defined as pain present in both sides of the body, above and below the waist and in axial body regions [6], and has a prevalence of about 11% in the adult population [7, 8]. In patients with rheumatoid arthritis (RA), CWP has been reported in one-third of the patients [9], but in other chronic rheumatic diseases such as spondyloarthritis (SpA), information regarding prevalence rates for CWP are limited.

SpA is a group of chronic rheumatic diseases with similar clinical features such as back pain, asymmetrical peripheral arthritis, enthesitis, and extra-articular manifestations, and includes―among others―AS and undifferentiated spondyloarthritis (USpA) [10]. In Sweden, all diagnoses in clinical practice are set in accordance with the International Classification of Diseases and Related Health Problems, Tenth Revision (ICD-10). The AS diagnosis confirms with both the 1984 modified New York criteria [11] and the more recent criteria for axial spondyloarthritis (axSpA) [12]. The USpA diagnosis refers to a less well studied and variable group of patients [10, 13], including patients with non-radiographic axial SpA and peripheral SpA, or a combination [12, 14, 15]. It has been argued that USpA, in some cases, can represent an early form of AS [16–19], and in a recent review, a relatively large group of patients with USpA (39.9%) developed into AS after 10 years [20]. Functional impairment, reduced quality of life, fatigue, reduced ability to work, anxiety, and depression, are well-known findings for patients with SpA in general [21–25].

There is an increased attention regarding difficulties of managing chronic pain in SpA in the clinic. But as to date, the few studies exploring chronic pain in SpA have mainly concentrated on patients with AS and pain corresponding to the more complex fibromyalgia (FM) syndrome [6], with prevalence estimates of FM varying between 4% and 15%, and with higher frequencies in women [26–29]. CWP can include pain from different origins, and be seen as a continuum, with FM representing the more severe form. Therefore, studies that aim to identify patients with early and less severe CWP could be important. Not only, for an accurate diagnosis, but most importantly, for optimal and early treatment of both the inflammatory disease, and a possible co-existing sensitization of the nervous system, requiring other treatment strategies.

The aim of the study was to assess differences in prevalence of self-reported CWP in a population-based cohort of patients diagnosed with AS or USpA, including differences between women and men.

## Methods

### The Skåne Health Care Register (SHCR)

The county of Skåne is the most southerly region of Sweden. All healthcare visits, for both inpatients, and outpatients, are registered in the Skåne Health Care Register (SHCR) using unique personal identification numbers. About one-eighth of the Swedish population is covered in the SHCR. Information on the healthcare provider, on the date of visit, and on ICD-10 diagnoses is included in the SHCR. More details of this register are given elsewhere [30, 31].

### Study population

This cross-sectional study made use of the population–based SpAScania cohort (*n* = 3711), which was identified through the SHCR during the period 2003–2007. For inclusion in the cohort, a diagnosis of SpA (with ICD-10 codes), was required to be registered, either by a rheumatologist or an internist on one occasion, or twice by any other physician in primary or secondary care on two separate occasions. A validation of the accuracy of the SpA diagnosis in the SHCR has previously been performed, with a valid diagnosis in 98% of the cases [30].

In 2009, a postal questionnaire was sent out to all patients in the cohort who were 18 years of age and over. Out of the 2162 patients who answered the survey (58%), 940 with a diagnosis of AS (ICD-10 code; M45.9) or USpA (ICD-10 codes; M46.0, M46.1, M46.8, M46.9) were included in the study. All patients with a diagnosis corresponding to psoriatic arthritis, IBD-related arthritis, or reactive arthritis were excluded. An analysis of non-responders within the large SpAScania cohort has previously been published [32]. This showed that patients with AS were more likely to respond, and that higher age predicted a higher response in men. There was an increased response with age also in women, except for women with AS, who tended to respond less with age.

### The questionnaire

The questionnaire consisted of several validated patient-reported outcome measures as described elsewhere [32]. Data on socio-demographics (age, sex), disease duration, pain (duration, distribution and intensity), fatigue, smoking habits (smoker/ever, smoker/never), body mass index (BMI), synthetic and biologic disease-modifying antirheumatic drugs (sDMARDs and bDMARDs), corticosteroids, and disease activity (according to the Bath Ankylosing Spondylitis Disease Activity Index, BASDAI) [33], were used in this study*.*

#### Details of pain

For assessing pain intensity, a numerical rating scale (NRS) was used, ranging from 0 (meaning no pain) to 10 (meaning worst possible pain). For pain to be considered chronic, it had to be persistent or recurrent for more than 3 months during the previous 12 months [6]. The overall question for musculoskeletal chronic pain was: “have you during the last twelve months experienced any aches or pains lasting more than three months?” To distinguish between chronic regional pain (CRP) and chronic widespread pain (CWP), a pain mannequin, with 18 predefined body regions, and explanatory names for each region, was used [7]. For CWP to be considered present, according to the 1990 American College of Rheumatology (ACR) criteria [6], pain was required to be marked (I) on both the left side and the right side of the body, (II) above and below the waist, and (III) in the axial regions (the cervical spine, anterior chest, thoracic spine, and lower back) of the mannequin. When the criteria for chronic pain were met, but not those for the widespread condition, patients were considered to have CRP. Patients, who answered “no” to the question defining chronic pain were regarded as having no chronic pain (NCP).

### Statistical analyses

Prevalence estimates for self-reported pain in AS and USpA, including age- and sex-adjusted prevalence, were calculated and differences in mean values were analysed with Student’s t-test. Differences in proportions were analysed with chi-square test. Multivariate logistic regression analysis was used to study the associations with (i) chronic pain (CRP and CWP) vs. NCP, (ii) and CWP vs. NCP/CRP, as dependent variables. Age, sex, SpA subgroup, smoking status, and BMI were all included in the analyses as independent variables, and thereby controlled for each other in the analyses. The multivariate logistic regression analyses were done with simple contrast to a reference group for each of the variables. Age- and sex- adjusted prevalence rates were adjusted by the direct method using the Swedish census population of 2009 as a standard population, to adjust for the differences in age and sex distribution in the AS and USpA groups. Analyses were performed using SPSS software version 20 for Windows (IBM Corp., NY, USA).

The study is reported according to the STROBE (STrengthening the Reporting of OBservational studies in Epidemiology) guidelines [34].

## Results

Patients with AS (*n* = 570) were older, with a mean age of 54.2 (SD 13.9) years vs. 49.1 (13.6) years, were more often men (65.6% vs. 41.6%), and had a mean disease duration that was twice as long (20.3 (SD 13.5) years vs. 10.3 (9.5) years) as that of patients with USpA (*n* = 370). There was no significant difference in pain intensity between AS and USpA, but patients with USpA reported having a higher number of pain regions than patients with AS (mean 5.3 (SD 4.9) vs. 4.5 (4.5), *p* = 0.019) (Table 1). In general, women reported having higher pain intensity (mean 4.2 (SD 2.5) vs. 3.5 (2.4), *p* ≤ 0.001) and a higher number of pain regions than men (mean 5.7 (SD 4.8) vs. 4.1 (4.6), *p* ≤ 0.001). Differences between women and men in the AS and USpA subgroups are presented in Table 2. The use of DMARDs and corticosteroids (solely or in combination) were reported by 50.4% of the patients. The frequency of sDMARDs and corticosteroids were similar between patients with AS and USpA, but more patients with AS reported using bDMARDs (AS: 19.3% vs. USpA: 14.6%, *p* = 0.005). In addition, self-reported use of sDMARDs and bDMARDs were similar between patients in the three pain groups (NCP, CRP, CWP), but more patients with CWP reported using corticosteroids compared to patients belonging to NCP or CRP (CWP: 16.8%, CRP: 5.2%, NCP: 7.7%, *p* < 0.001).

**Table 1:** Clinical characteristics of the patients with AS and USpA (*n* = 940)

	AS	USpA	
Variables	*n* = 570	*n* = 370	*p-value*
Age, years	54.2 (13.9)	49.1 (13.6)	*≤ 0.001*
Women/Men, n (%)	196/374 (34.4/65.6)	216/154 (58.4/41.6)	*≤ 0.001*
Disease duration, years	20.3 (13.5)	10.3 (9.5)	*≤ 0.001*
Pain intensity (0–10)	3.7 (2.7)	4.0 (2.5)	*0.081*
Fatigue (0–10)	4.4 (2.7)	4.7 (2.8)	*0.222*
Pain regions	4.5 (4.6)	5.3 (4.9)	*0.019*
Smoking ever %	53.7	38.6	*≤ 0.001*
BMI	25.9 (4.0)	25.6 (4.3)	*0.256*
BASDAI (0–10)	3.9 (2.2)	4.2 (2.2)	*0.031*

Values are given in mean (SD) unless otherwise indicated

*AS* ankylosing spondylitis, *USpA* undifferentiated spondyloarthritis, *BMI* body mass index, *BASDAI* the Bath Ankylosing Spondylitis Disease Activity Index

**Table 2:** Clinical characteristics of women and men with AS (*n* = 570) and USpA (n = 370)

	AS			USpA		
Variables	Women	Men	*p-value*	Women	Men	*p-value*
	*n* = 196	*n* = 374		*n* = 216	*n* = 154	
Age, years	52.2 (14.2)	55.2 (13.6)	*0.016*	49.1 (13.8)	49.0 (13.4)	*0.924*
Disease duration, years	16.7 (12.4)	22.0 (13.7)	*≤0.001*	9.7 (8.9)	11.1 (10.3)	*0.207*
Pain intensity (0–10)	4.2 (2.6)	3.4 (2.4)	*0.001*	4.2 (2.5)	3.6 (2.4)	*0.022*
Fatigue (0–10)	5.0 (2.9)	4.1 (2.6)	*≤0.001*	5.0 (2.9)	4.2 (2.6)	*0.010*
Pain regions	5.2 (4.7)	4.2 (4.6)	*0.022*	6.2 (4.9)	4.0 (4.5)	*≤0.001*
Smoking ever, %	48.0	56.7	*0.054*	38.4	39.0	*0.878*
BMI	25.0 (4.4)	26.4 (3.7)	*≤0.001*	25.4 (4.7)	25.9 (3.7)	*0.272*
BASDAI (0–10)	4.2 (2.3)	3.7 (2.2)	*0.033*	4.6 (2.1)	3.8 (2.2)	*0.001*

Values are given in mean (SD) unless otherwise indicated

*AS* ankylosing spondylitis, *USpA* undifferentiated spondyloarthritis, *BMI* body mass index, *BASDAI* the Bath Ankylosing Spondylitis Disease Activity Index

Five per cent (53/940) of the patients could not be categorized into any of the pain groups (NCP, CRP, or CWP) due to missing responses on the chronic pain question and the pain mannequin, leaving 536 patients with AS and 351 patients with USpA. The patients not responding to the chronic pain questions had a mean age of 55.5 (SD 12.9) years (AS 57.5 (SD 12.8) vs. USpA 52.0 (SD 12.7), *p* = 0.137), and were more often men (62.3%). In addition, 64.2% of the non-responders had AS.

### The prevalence of CRP and CWP

The one-year period prevalence of CRP was 19.4% (17.7% with AS vs. 21.9% with USpA), and that of CWP 46.9%. CWP was significantly more common in USpA than in AS (49.3% vs. 45.3%, *p* = 0.033). CWP was also more common in women than in men, with a prevalence for the total SpA group of 54.1% vs. 41.2% (*p* ≤ 0.001), while there was an equal sex distribution regarding CRP (Table 3).

**Table 3:** Prevalence of pain (%) in men and women, for AS and USpA respectively, based on the pain groups: no chronic pain (NCP), chronic regional pain (CRP), and chronic widespread pain (CWP)

	AS				USpA			
	Women	Men	Total		Women	Men	Total	
	*n* = 184	*n* = 352	*n* = 536	*p-value*	*n* = 208	*n* = 143	*n* = 351	*p-value*
NCP	30.4	40.3	36.9		22.1	38.5	28.8	
CRP	17.9	17.6	17.7		21.6	22.4	21.9	
CWP	51.6	42.0	45.3	*0.059* ^*a*^	56.2	39.2	49.3	*0.002* ^*a*^

Statistical comparison by chi square test

*AS* ankylosing spondylitis, *USpA* undifferentiated spondyloarthritis

^a^for whole table with NCP, CRP and CWP

Pain intensity was not significantly different between women and men in patients reporting CRP (mean (SD) 4.0 (2.3) vs. 3.5 (2.0), *p* = 0.095) or in those reporting CWP (5.1 (2.3) vs. 5.0 (2.2), *p* = 0.622).

### Age- and sex-adjusted prevalence rates for CWP

Assuming that those who did not answer the pain duration and distribution questions had NCP, this would give a minimum age- and sex-adjusted prevalence of CWP for the total group (*n* = 940) of 44.9% (95% Cl 39.1–50.7), being higher for women (53.5%, 95% Cl 43.8–63.1) than for men (36.2%, 95% Cl 29.9–42.6). For patients with AS, the minimum age- and sex-adjusted prevalence of CWP was 42.7% (95% Cl 34.4–51.0) as compared to 47.8% (95% Cl 37.4–58.2) in patients with USpA, and this was mainly explained by the higher prevalence of CWP in women with USpA (57%, 95% Cl 42.4–71.6) than in women with AS (50.4%, 95% Cl 35.4–65.3).

### Variables associated with chronic pain

In the multivariate logistic regression analyses, female sex was associated with chronic pain vs. NCP (odds ratio (OR) 1.78), and with CWP vs. NCP/CRP (OR 1.70), controlled for age, SpA subgroup (AS or USpA), smoking status, BMI, and disease duration. A higher BMI was associated with chronic pain (OR 1.05), and CWP (OR 1.05), while being an ever-smoker was associated with CWP only (OR 1.44). Belonging to a specific subgroup (AS vs. USpA), or experiencing a longer disease duration was not associated with either chronic pain, or CWP, when controlling for all other variables (Table 4).

**Table 4:** Results from the logistic regression analysis with odds ratios (OR) and 95% confidence interval for having (i) chronic pain vs. NCP, and (ii) CWP vs. NCP or CRP. The independent variables were all included, and thereby controlled for each other in the analyses

	Chronic pain (*n* = 773)		CWP (*n* = 767)	
	OR (95% Cl)	*p-value*	OR (95% Cl)	*p-value*
Sex				
Men	1		1	
Women	1.91 (1.37–2.67)	*≤0.001*	1.70 (1.25–2.32)	*0.001*
Age	1.01 (1.00–1.03)	*0.080*	1.01 (1.0–1.03)	*0.088*
Diagnosis				
AS	1		1	
USpA	1.41 (0.99–2.00)	*0.055*	1.11 (0.80–1.53)	*0.546*
Smoking				
Never	1		1	
Ever	1.33 (0.97–1.83)	*0.081*	1.44 (1.07–1.95)	*0.016*
BMI	1.05 (1.10–1.10)	*0.022*	1.05 (1.01–1.09)	*0.010*
Disease duration	0.99 (0.97–1.01)	*0.184*	1.00 (0.98–1.01)	*0.656*

*AS* ankylosing spondylitis, *USpA* undifferentiated spondyloarthritis, *NCP* no chronic pain, *CRP* chronic regional pain, *CWP* chronic widespread pain, *BMI* body mass index

## Discussion

In this study, we found a high prevalence of self-reported chronic pain in both AS and USpA. CWP was present in half of the patients with USpA, and just slightly less in patients with AS, and was overall more common in women. About one-fifth of the patients with either AS or USpA reported having CRP, with no differences between women and men. Female sex, a higher BMI, and being an ever-smoker were associated with CWP in contrast to NCP/CRP, while diagnosis (AS or USpA) and disease duration were not. These findings can complicate the evaluation of disease activity and response to treatment in patients with AS and USpA, and emphasise the need of an early and thorough clinical examination, including not only evaluation of inflammation, but also an accurate and careful pain analysis.

The results from the present study are difficult to compare in relation to previous research on SpA, since to our knowledge, there have been no studies on the prevalence of CWP in AS and USpA without limiting it to FM. However, the prevalence of CWP in AS and USpA was clearly higher than in the general population [7, 8], and also, higher than in a previous report on the prevalence of CWP in RA (34%) [9]. We used the same pain mannequin and definition of chronic pain as the two previous Swedish studies [7, 9].

Previous research has found that it is important to consider that the evaluation and diagnosis of SpA, particularly in women, can be delayed [35] and complicated by the presence of CWP. Symptoms such as chronic back pain, stiffness, and fatigue are common in patients with both SpA and FM, and can be interpreted, as indicating an increase in disease activity [26, 27, 36, 37]. Also difficulties in distinguishing fibromyalgia tender points and enthesitis sites in SpA have been reported [38], with an overlap of about 30% between the inflammatory back pain (IBP) criteria and the FM criteria. Even though we could not examine for tender points or enthesitis and the study population was different, it is important to acknowledge that CWP can include pain from different causes. CWP is a prerequisite for FM, and patients with CWP could already have FM, or be at risk of developing FM at a later date [39]. Interestingly, the opposite scenario has also been found, where almost half of the patients, particularly women, were incorrectly diagnosed with FM instead of an inflammatory rheumatic condition [40]. With the above in mind, it is important to stress the fact that a thorough evaluation, including a pain assessment to identify signs of a widespread nature, is important in all patients who report prolonged and increased pain, and other symptoms such as fatigue and poor quality of life. In addition, the origin of pain is important when it comes to treatment, but the presence of enthesitis does not exclude a co-existing sensitivity in the pain system―which is why individualized pain management, including both non-pharmacological and pharmacological treatment, is emphasized.

The women in our study had a higher prevalence of CWP, and CWP was associated with female sex in the logistic regression analysis. These findings are in accordance with the results of previous studies that have investigated FM in SpA [26–29, 36], and with a recent review [41], reporting evidence of a higher risk of developing chronic pain in women than in men. In the same review, some evidence―although inconclusive―was found that women experience more severe clinical pain than men, and this was contributed to multiple bio-psychosocial mechanisms including hormones, neurochemistry, social roles, and coping mechanisms [41]. In the present study, no sex-related difference in the prevalence rates of CRP were found, and although the prevalence of CWP was lower in men than in women, men with AS and USpA reported a high prevalence of CWP, compared to men in the general population [7]. Interestingly, we also found that pain intensity was not significantly different in women and men, when studied separately in those with CRP or CWP, and that an overall higher pain intensity in women was due to the higher prevalence of CWP in women. These findings are new, and highlight the fact that it is important to be aware of and recognize a concomitant CWP also in men with SpA.

In agreement with earlier studies in the general population [42, 43], CWP was also associated with being an ever-smoker and having a higher BMI. People with obesity can have a low-grade systemic inflammation, due to the production of pro-inflammatory cytokines and adipokines in the white adipose tissue [44]. The association between obesity and chronic pain has accordingly, been reported to partly be mediated by pro-inflammatory cytokines, but biomechanical and structural changes, mood, poor sleep, lifestyle factors and personal factors have also been found to be important mediators [45].

We found no association between SpA disease duration and chronic pain in this study. One reason for this may be that personal or other factors are more important than disease duration when it comes to developing chronic pain, and especially CWP.

Strengths of the present study were that patients from both primary and specialist health care were included, and the relatively large sample size. Another strength was that the instruments represent different dimensions of pain (duration, distribution, and intensity), and are commonly used and validated [7, 46, 47]. There were also some important limitations of the study. One was the low response rate, even though this was comparable to that in other population-based surveys [7, 42, 43]. Another limitation was that the patients were identified by their clinical ICD-10 diagnosis so we cannot be certain how many patients with USpA that would be categorized as having axial or peripheral SpA. However, in this study patients with psoriatic arthritis, IBD-related arthritis, or reactive arthritis were excluded, and 65% of the patients with USpA (M46.0-1 and M46.8-9) reported current chronic axial involvement, possibly representing an early form of AS. Moreover, the questionnaire lacked information on other comorbid diseases that could have impact on chronic pain. Also, information regarding socio-demographic variables would have been interesting with regard to CWP. The self-reported data regarding medication should due to the large proportion of missing data, be interpreted with care. Finally, the cross-sectional design makes us unable to draw conclusions as to the causality of the associations detected. In future research, longitudinal studies will be important to help us gain a better understanding of predictive factors for development of CWP in patients with SpA.

## Conclusion

We found a high prevalence of concomitant CWP in patients with AS or USpA, with an even higher prevalence in women, but with no difference in the intensity of pain in women and men who experienced CWP. The results highlight the importance of a thorough pain analysis included in the clinical examination, to identify patients with high and/or increasing pain levels and multiple pain regions. It may also guide appropriate and individualized treatment decisions, including non-pharmacological and pharmacological treatment options.

## Abbreviations

ACR: American College of Rheumatology
AS: Ankylosing spondylitis
ASAS: The Assessment of Spondyloarthritis Society
BASDAI: The Bath Ankylosing Spondylitis Disease Activity Index
bDMARDs: Biologic disease-modifying antirheumatic drugs
BMI: Body mass index
CRP: Chronic regional pain
CWP: Chronic widespread pain
FM: Fibromyalgia syndrome
ICD-10: The International Classification of Diseases and Related Health Problems, Tenth Revision
NCP: No chronic pain
NRS: Numerical rating scale
OR: Odds ratio
RA: Rheumatoid arthritis
sDMARDs: Synthetic disease-modifying antirheumatic drugs
SHCR: The Skåne Health Care Register
SpA: Spondyloarthritis
USpA: Undifferentiated spondyloarthritis
